# Endocytosis of IgG, Desmoglein 1, and Plakoglobin in Pemphigus Foliaceus Patient Skin

**DOI:** 10.3389/fimmu.2019.02635

**Published:** 2019-11-12

**Authors:** Dyah A. M. Oktarina, Ena Sokol, Duco Kramer, Marcel F. Jonkman, Hendri H. Pas

**Affiliations:** ^1^Department of Dermatology, Centre for Blistering Diseases, University Medical Centre Groningen, University of Groningen, Groningen, Netherlands; ^2^Department of Dermatology and Venereology, Faculty of Medicine, Public Health and Nursing, Universitas Gadjah Mada, Yogyakarta, Indonesia

**Keywords:** pemphigus foliaceus, desmoglein 1, desmosomes, acantholysis, endocytosis

## Abstract

Pemphigus foliaceus (PF) is one of the two main forms of pemphigus and is characterized by circulating IgG to the desmosomal cadherin desmoglein 1 (DSG1) and by subcorneal blistering of the skin. The pathomechanism of blister formation in PF is unknown. Previously we have shown that PF IgG induces aggregation of DSG1, plakoglobin (PG), and IgG outside of desmosomes, what in immunofluorescence of PF patient skin visualizes as a granular IgG deposition pattern with a limited number of coarse IgG aggregates between cells. Here we have investigated the fate of these aggregates in skin and found that these are cleared by endocytosis. We performed double immunofluorescence staining on snap-frozen skin biopsies of six PF patients for the following molecules: IgG, the desmosomal proteins DSG1 and DSG3, desmocollins 1 and 3, PG, desmoplakin and plakophilin 3, and for the endosomal marker early endosomal antigen 1 and the lysosomal markers cathepsin D and lysosomal-associated membrane protein 1. Endosomes were present in all cells but did not make contact with the aggregates in the basal and suprabasal layers. In the higher layers they moored to the aggregates, often symmetrically from two adjacent cells, and IgG, DSG1, and PG were taken up. Finally these endosomes became localized perinuclear. Endocytosis was only observed in perilesional or lesional skin but not in non-lesional skin. Older immunoelectron microscopic studies have suggested that in PF skin endocytosis of detached desmosomes takes place but we found no other desmosomal proteins to be present in these endosomes. Double staining with cathepsin D and LAMP-1 revealed no overlap with IgG, DSG1, or PG suggesting that lysosomes have no role in the clearing process. Collectively, our results show that endocytosis is part of the pathogenic process in PF but that no detached desmosomes are taken up but instead the deposited IgG is taken up together with DSG1 and PG.

## Introduction

Pemphigus is an autoimmune blistering disease of the skin and mucous membranes, which is characterized by a loss of cell-cell adhesion, known as acantholysis. Pemphigus foliaceus (PF) is one of the two major forms of pemphigus and it is characterized by blistering of the skin only and immunologically by circulating autoantibodies against desmoglein 1 (DSG1) (1, 2). DSG1 is a glycoprotein of desmosomes and is expressed through all the epidermal layers. Desmosomes are intercellular adhesion structures and are build-up of transmembrane desmosomal cadherins desmogleins and desmocollins (DSC), that by heterophilic interaction directly interconnect two opposite half-desmosomes, and intracellular desmosomal plaque proteins including plakoglobin (PG), desmoplakin (DP), and plakophilins (PKP) (3–6). The intracellular proteins connect the cytoplasmic tails of the desmosomal cadherins to the intermediate keratin filaments thereby providing strength to the tissue. The pathomechanism of the loss of cell-cell adhesion in PF is not exactly known and compared to PV only few studies have addressed it. It is clear that the anti-DSG1 IgG is the pathogenic factor that induces acantholysis as injection of PF IgG causes skin blistering in mice and in an *ex vivo* living human skin model (7–9). Injecting of PF IgG in mice also results in activation of p38MAPK and an inhibitor to p38MAPK abolishes this blistering (7). Waschke et al. showed that PF IgG reduces Rho A activity and in an *ex vivo* human skin model PF induced blistering could be abrogated by Rho A activation (10). Recent research by Walter et al. found that anti-DSG3 IgG and anti-DSG1 IgG led to activation of different signaling pathways. While both activated p38MAPK, anti-DSG3 activated Src while in contrast anti-DSG1 activated ERK, indicating that the pathomechanisms between PV and PF might differ (11). Steric hindrance i.e., obstruction of DSG transinteraction is also considered as a possible pathomechanism. Based on single molecule atomic force measurements and by laser trapping of surface- bound DSG1-coated microbeads Waschke et al. however found no evidence for this (12). We have shown that in the anti-DSG1 IgG induces a shift in distribution of DSG1 and PG in PF patient skin (8). In healthy human skin staining for DSG1 shows an evenly distributed signal over the cell membranes in line with a desmosomal distribution while instead in PF skin, especially in the lower epidermal layers, DSG1 is present in coarse clusters that also contain PG and IgG but no other desmosomal proteins. These clusters can be induced by bivalent PF IgG in the *ex vivo* living human skin model, but not by monovalent Fab fragments of this same IgG which suggest that crosslinking of DSG molecules underlies cluster formation (8, 9). Recently further evidence was provided that the polyvalence of bivalent PF IgG is responsible for clustering. A mixture of non-pathogenic PF monoclonal antibody (mAb) and pathogenic PF mAb is needed to induce clusters, but only pathogenic PF mAb is able to induce the loss of cell-cell adhesion (13). The clusters in PF skin contain DSG1 and PG, but no other desmosomal components which suggests that desmosomes become depleted of DSG1 (8). In skin where IgG has induced clustering of DSG1 desmosomes become reduced in size and number (14). The reduction in size of the desmosomes can be induced in *ex vivo* living human skin by both pathogenic or non-pathogenic PF mAb, while their mixture enhances this effect (13).

Tada and Hashimoto (15) studied patient skin by immunoelectron microscopy and found what they called curvicircular cytoplasmic bodies in PF but not in PV skin. These were present in the higher, but not in the lower layers of the epidermis. These structures labeled positive for DSG1, PG, IgG, and connexin 43 (CNX43), and did not contain attachment plaques or inserted tonofilaments. These were hypothesized to be internalized IgG-bound desmosome-gap-junction complexes that transformed into curvicircular structures. In 1999, Iwatsuki et al. described cytoplasmic vesicles in acantholytic keratinocytes that labeled positive for DSG1 and seemed to contain detached desmosomes. We recently described the curvicircular structures to be double membrane structures, that may look intracellular but in fact seem the result of closely sealed (40 nm width) neighboring membranes (www.nanotomy.org). In perilesional skin they are present in the lower epidermal layers and they spread upwards to higher layers in lesional skin (16). We have speculated that these double membrane structures likely are the DSG1-PG-IgG clusters seen by light microscopy.

In order to further unravel the pathomechanism of PF we address in the present the faith of DSG1-PG-IgG clusters and investigate if endocytosis of DSG1 takes place in PF patient skin.

## Materials and Methods

### Patient Biopsies

We included 6 PF patients; from these 5 had a biopsy from lesional skin, 1 from perilesional skin, and from three patients we also had a concomitant biopsy from healthy skin.

The diagnosis in all six patients had been established on clinical criteria and laboratory investigation, including histology, positive immunofluorescence of skin (DIF), positive binding of serum IgG (1/40 dilution) to monkey esophagus in an epithelial cell surface (ECS) pattern, and serum anti-DSG1 antibodies by MESACUP-2 ELISA test for anti-DSG1 (MBL, Japan). The skin specimens had been immediately frozen in liquid nitrogen and stored at −80°C. Skin obtained from breast reduction was used as healthy control skin. According to national regulations this type of retrospective non-interventional study on leftover tissue from diagnostics does not need approval from the local medical ethical committee.

### Immunofluorescence Microscopy

The procedure for immunofluorescence staining and image collection has been described in detail before (8). For visualization of adhesion molecules we used the following monoclonals: DSG1-P23 and DSG1-P124 (DSG1 ectodomain), 27B2, DG3.10, 18D4 and B-11 (DSG1 endodomain), DSG-G194 (DSG3), U100 (DSC1), U114 (DSC3), 15F11 (PG), DP2.15 (DP), and PKP3-270.6.2 (PKP3). Early endosomal antigen (EEA1) was stained with 14/EEA1, cathepsin D (CTS D) was stained with CTD-19, LAMP-1 with H4A3, and connexin 43 with 4E6.2. Double staining of IgG and adhesion molecules IgG was performed with fluoresceinthiocyanate (FITC)-conjugated Fcγ-specific goat F(ab')2 anti-human IgG (Protos Immunoresearch, Burlingame, CA, U.S.A) and Alexa 568-conjugated goat anti-mouse IgG (Molecular Probes Eugene, OR, U.S.A) as secondary steps. For double staining with two different mouse monoclonals we used Zenon® Mouse IgG Labeling Kits Alexa Fluor®488 and Alexa Fluor®568 (Molecular Probes, Invitrogen, USA) by following technical protocols. Supplementary Table 1 contains details on antibodies used.

## Results

### Endosomal Uptake Takes Place in the Upper Layers of Lesional Pemphigus Foliaceus Skin

**Figure 1:**
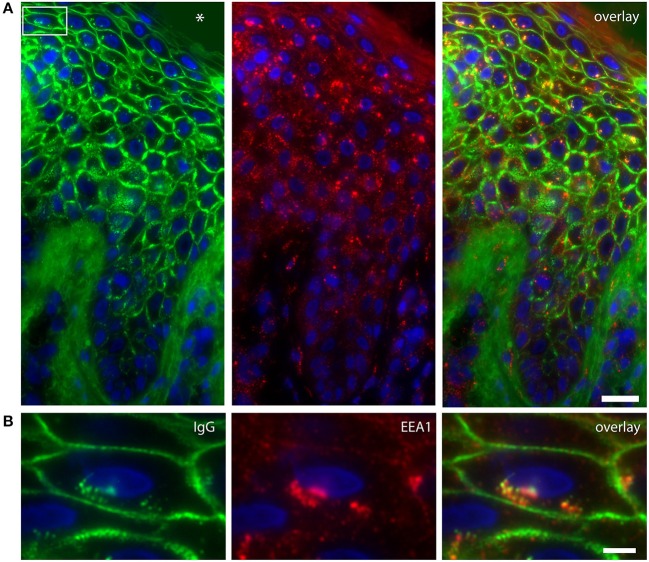
Colocalization of IgG and endosomes in lesional PF patient skin. **(A)** IgG (green) and EEA1 (red) colocalize in the higher skin layers (right panel). The white box depicts the cell shown in **(B)**. The blister cavity is indicated by an asterix. White bar: 25 μm. **(B)** Detail showing the perinuclear localization of IgG. White bar: 5 μm.

**Figure 2:**
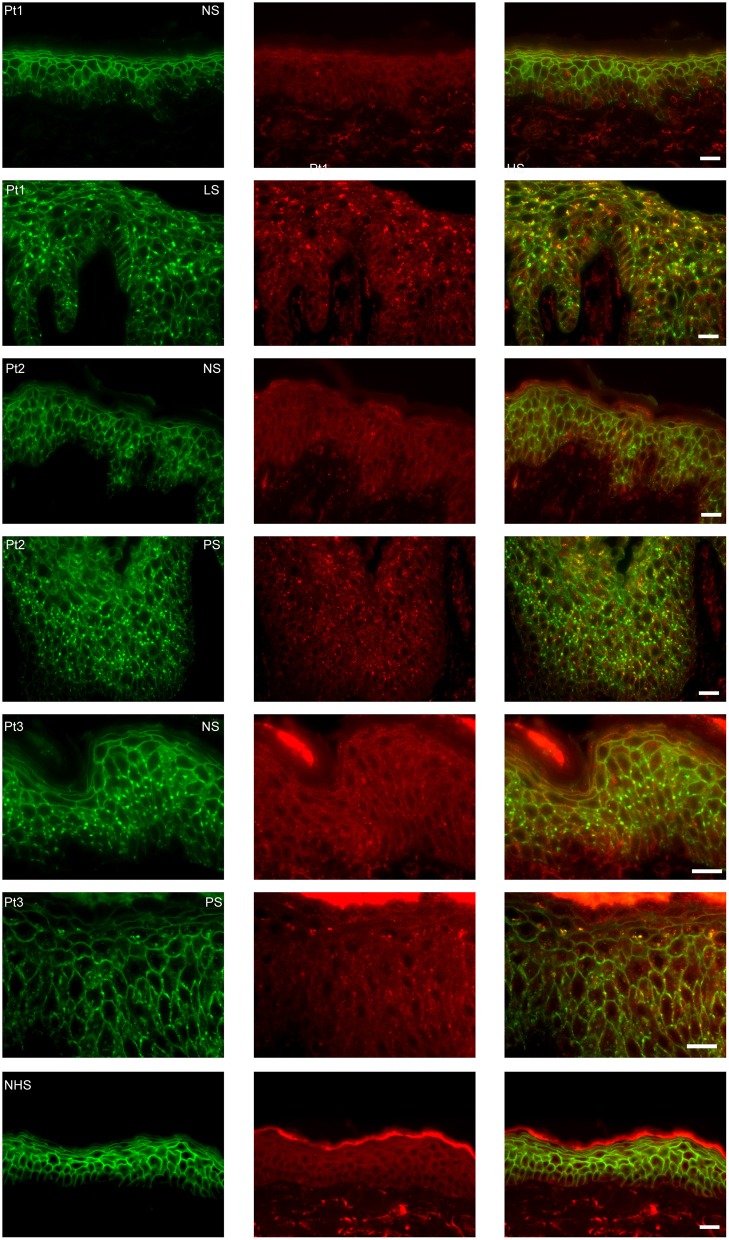
Endocytosis from clusters is not present in non-lesional skin. The images are from three patients of whom we had biopsies of non-lesional skin next to biopsies of perilesional or lesional skin. As control healthy normal human skin is used. In green plakoglobin and in red early endosomal antigen. The panels to the right are overlays. Pt, patient; NS, non-lesional skin; PS, perilesional skin; LS, lesional skin; NHS, normal human skin. White bar is 20 μm.

In order to examine the fate of DSG1, PG, and IgG clusters, we double stained the perilesional and lesional biopsies for EEA1 and IgG. In 4 of the 6 biopsies EEA1 colocalized with IgG demonstrating that IgG is taken up in endosomes. This is most prominent in the higher epidermal layers, next to and beneath the blister (Figure 1A). In more differentiated cells these endosomes are largely localized perinuclear (Figure 1B). In the 3 biopsies of non-lesional skin of our PF patients no endocytosis was observed. Figure 2 compares endocytosis of PG in non-lesional skin with perilesional or lesional skin of the same patients. In normal healthy human skin no overlap of EEA1 and PG was observed (Figure 2).

### Endosomes Contain Desmoglein 1 and Plakoglobin but No Other Desmosomal Proteins

**Figure 3:**
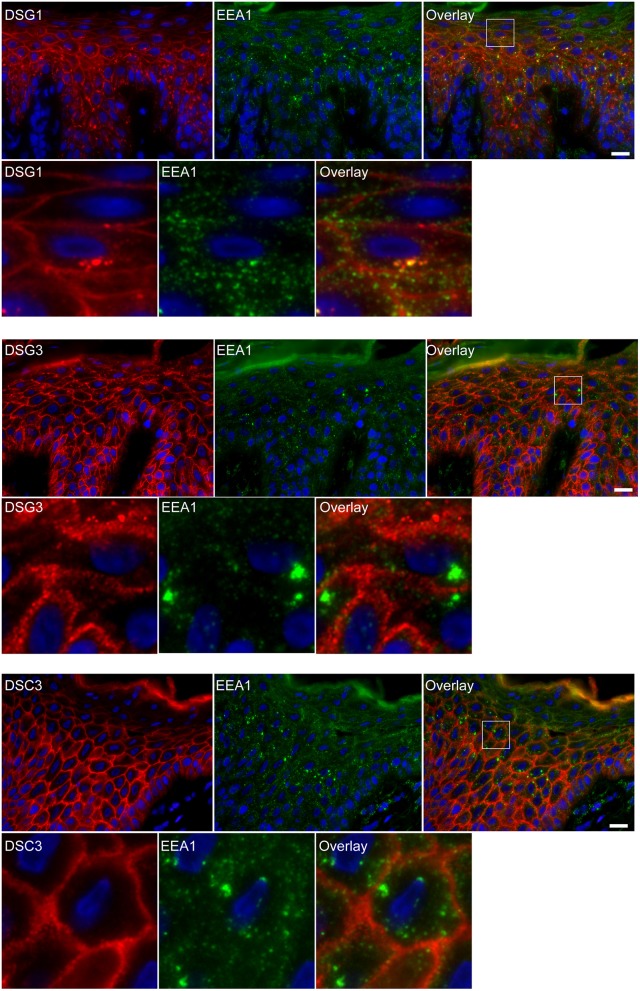
Double staining shows colocalization of EEA1 with desmoglein 1 but not with desmoglein 3 and desmocollin 3. The upper panels show the overviews, the lower panels shows the details from the white boxes in the upper panels. White bar is 20 μm.

**Figure 4:**
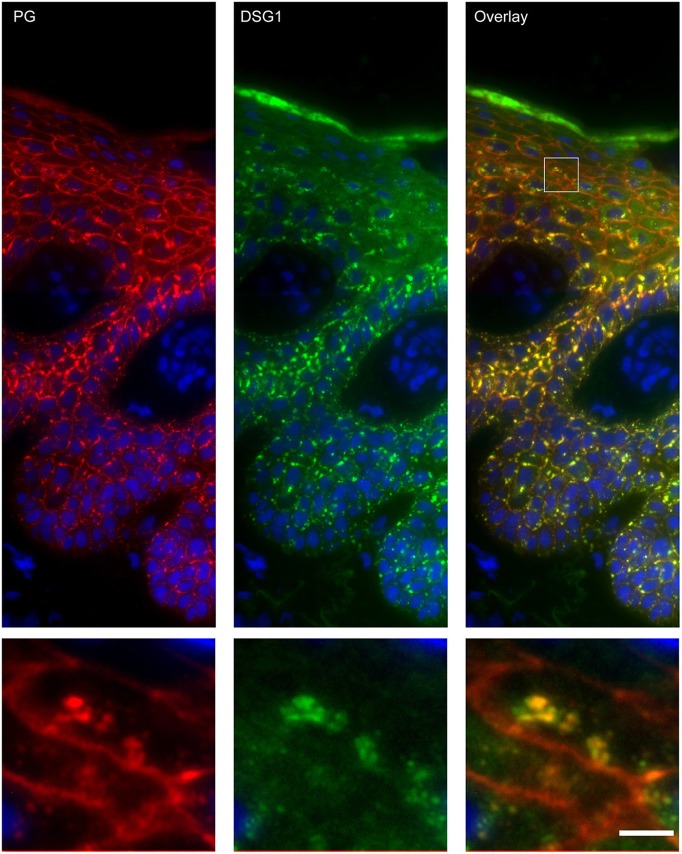
Double staining of desmoglein 1 and plakoglobin 1 shows that these are both present in the endosomes. The upper panel shows the overview, the lower panel shows the detail from the white box in the upper panel. White bar is 5 μm.

**Figure 5:**
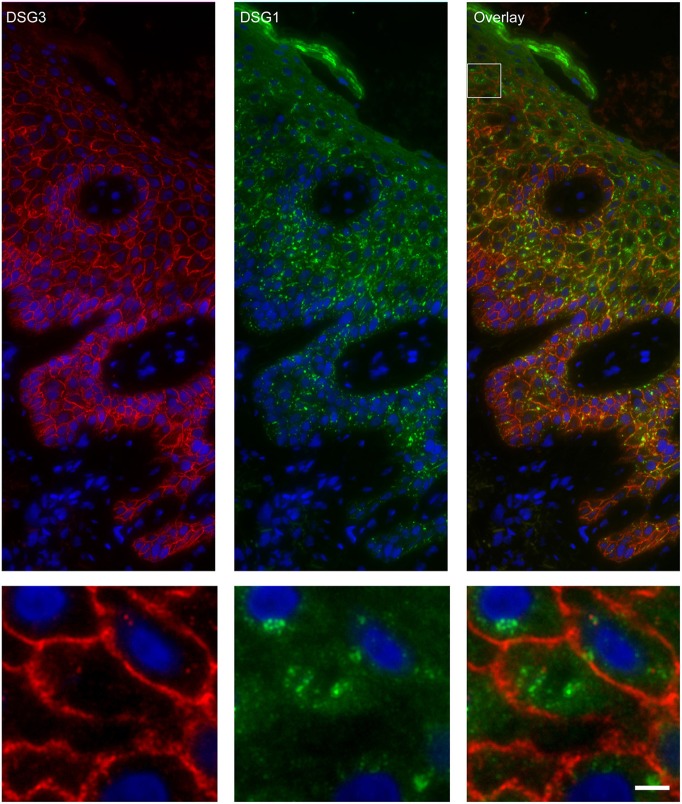
Double staining of desmoglein 1 and desmoglein 3 shows no overlap in endosomes. The upper panel shows the overview, the lower panel shows the detail from the white box in the upper panel. White bar is 5 μm.

**Figure 6:**
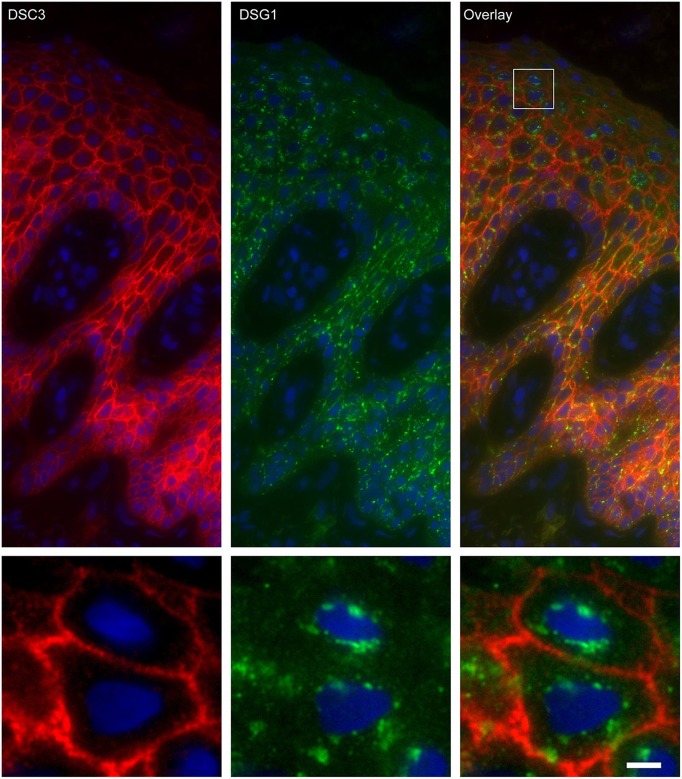
Double staining of desmoglein 1 and desmocollin 3 shows no overlap in endosomes. The upper panel shows the overview, the lower panel shows the detail from the white box in the upper panel. White bar is 5 μm.

**Figure 7:**
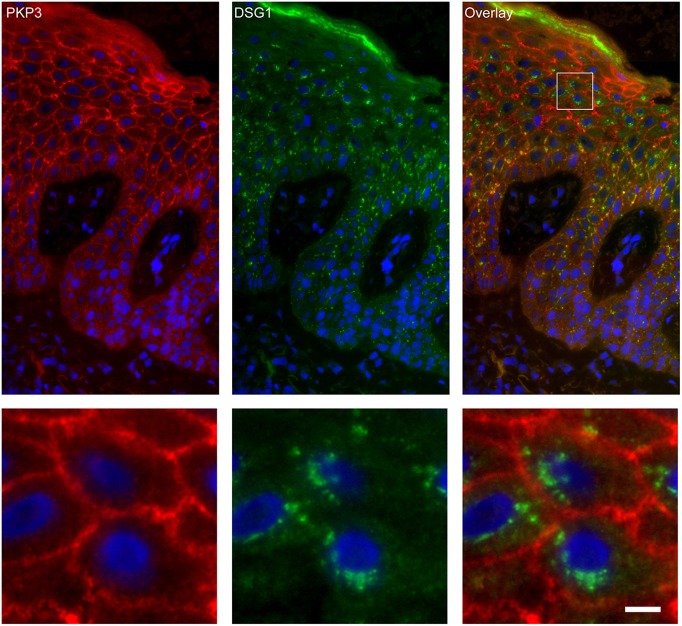
Double staining of desmoglein 1 and plakophilin 3 shows no overlap in endosomes. The upper panel shows the overview, the lower panel shows the detail from the white box in the upper panel. White bar is 5 μm.

**Figure 8:**
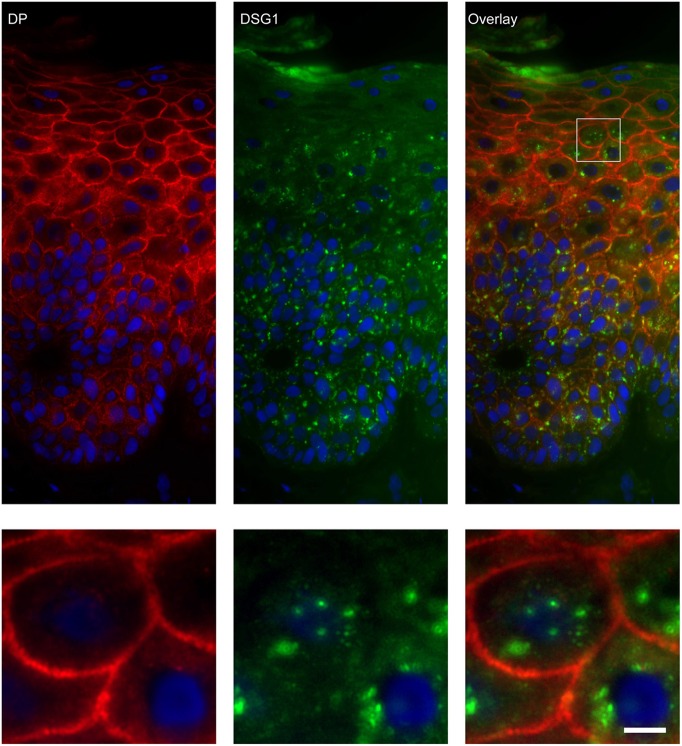
Double staining of desmoglein 1 and desmoplakin shows no overlap in endosomes. The upper panel shows the overview, the lower panel shows the detail from the white box in the upper panel. White bar is 5 μm.

Apart from IgG and PG also DSG1 is present in the same endosomes. In order to examine if other desmosomal components are also endocytosed we examined the localization of other desmosmal proteins in relation to the EEA1/DSG1/PG clusters. No overlap was found. Figure 3 shows colocalization of EEA1 with DSG1 but not with DSG3 and DSC3. Where DSG1 and PG (Figure 4) colocalized in the intercellular vesicles no co-localization of DSG1 with other desmosomal proteins was observed (Figures 5–8). The absence of desmosomal proteins as for instance DP in the clusters suggests that extra-desmosomal rather than desmosomal DSG1 is endocytosed.

### Endosomal Uptake Starts From the IgG Clusters

**Figure 9:**
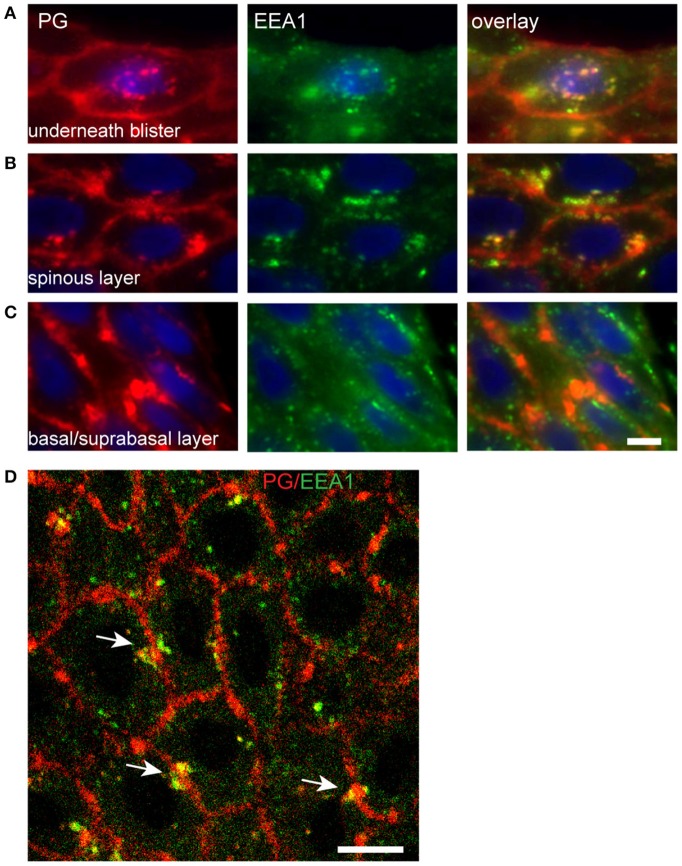
Endocytosis of plakoglobin starts in the spinous layer. Lesional PF skin stained for PG (red) and EEA1 (green). **(A)** Below the blister cavity; **(B)** spinous layer; **(C)** basal/suprabasal layer. Note the colocalization of PG and EEA1 in the spinous layer and below the blister cavity. White bar: 5 μm. **(D)** Confocal microscopy of lesional PF skin stained for PG (red) and EEA1 (green). White arrows indicate symmetrical endocytosis into neighboring keratinocytes. White bar: 10 μm.

In the basal layer endosomes do not contact the IgG clusters (Figure 9C). Upwards in the spinal layer endosomes moor on to the clusters and internalization starts to take place (Figure 9B). Then finally they become localized perinuclear (Figure 9A). Confocal microscopy shows that the endocytosis from the cluster takes place symmetrically in both cells that share the cluster (Figure 9D, white arrows).

### Endosomes Do Not Contain Connexin 43 and Do Not Fuse With Lysosomes

**Figure 10:**
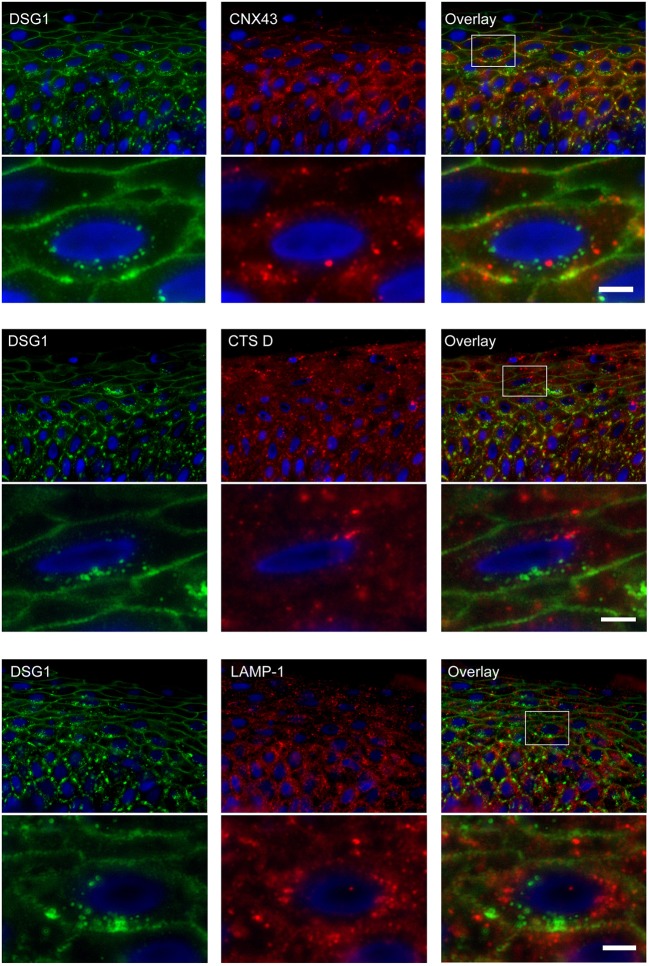
No colocalization of endocytosed desmoglein 1 with connexin 43 and lysosomes in PF skin. Lesional PF patient skin stained for DSG1 and CNX43, upper panel overview with detail underneath, DSG1 and CTS D, middle panel overview with detail underneath, and DSG1 and LAMP-1, lower panel overview with detail underneath. The location of the detailed area is indicated by a white box in the overview. No colocalization is observed. White bar: 5 μm.

Double staining of DSG1 and CNX43 showed that both were present in intracellular vesicles but these did not colocalize, implying they originate from different mechanisms (Figure 10). Surprisingly CTS D and LAMP-1 did also not colocalize with DSG1 indicating that lysosomes are not a destiny of the IgG-DSG1-PG clusters (Figure 10).

## Discussion

In this study we demonstrate that the clustered deposits of IgG in PF patient skin are cleared by endocytosis together with the co-clustered components DSG1 and PG (8). Although clusters are present in the basal epidermal layers active endocytosis starts in the spinous layer. Endocytosis is symmetrical in the way that in takes simultaneously place in both cells that share the cluster and progresses in more differentiated keratinocytes toward the cell nucleus.

Tada and Hashimoto suggested that binding of PF autoantibodies induce internalization of intact desmosomes and gap junctions in form of curvicircular structures that contained IgG, DSG1, PG, and CNX43 (15). Our double labeling results however show that CNX43 is present in separate organelles that are likely internalized annular gap junctions (17). Furthermore we did not find internalizations that contained other desmosomal components meaning that endocytosis of intact or half-desmosomes does not take place in PF skin as suggested before (18). Cirillo et al. demonstrated internalization of DSG1 upon incubation with PF patient sera in a monolayer of cultured cells (19). Cultured cells have characteristics of cells from the basal epidermal layer and no internalization of DSG1 was seen in the basal cells in any of PF biopsies. Therefore, it seems that conclusions reached with cell models have to be interpreted with care.

In contrast with PF, PV was extensively studied in cultured cells and it is shown that PV IgG upon incubation is taken up by endocytosis together with DSG3 and PG (20–23). After 3 h the IgG-DSG3-PG complex colocalized with lysosomes which suggests that cultured cells degrade internalized IgG. However, in PF patient skin we could not find evidence for such a degradation pathway. This suggests that internalized PF-IgG containing endosomes remain in the cell and are finally lost by desquamation. Furthermore in cell monolayer experiments it was shown that upon incubation PV IgG rapidly depletes soluble DSG3 from the membrane followed later by disappearance of desmosomal DSG3 (20, 21). Jennings et al. showed that prior to its internalization insoluble DSG3 is reorganized together with other desmosomal proteins into structures that are perpendicular to the cell membranes that were named linear arrays (23). Stahley et al. by super resolution microscopy observed structures in mucocutaneous PV skin they believed to be the linear arrays from which endocytosis of insoluble DSG3 takes place (24). Although we used simple immunofluorescence microscopy our data do not in any way suggest that such arrays are present in PF skin. Therefore, PV and PF pathogenesis might differ in the way IgG and DSG1 are endocytosed. An indication for this is that in PV skin colocalization of IgG and EEA1 was present in multiple small puncta near the cell membranes while we observed in PF skin that colocalization of IgG and EEA1 is in very large endosomes and only in the upper epidermal layers (24). Furthermore unlike in PV skin endocytosis in PF skin is restricted to the clusters that are induced by the anti-DSG1 IgG only.

Taken together we propose the following sequence of the events in PF patient skin. PF IgG binding to non-desmosomal DSG1 induces clustering and reorganization of non-desmosomal DSG1 together with PG which is bound to the cytoplasmic tail of DSG1. As other molecules as DP are absent desmosomal DSG1 is likely not included in these clusters. This causes lack of DSG1 in desmosomal turnover resulting in depletion of DSG1 from desmosomes and reduction of their size. Reorganized DSG1-PG-IgG are trapped into clusters which are likely the double membrane structures seen by electron microscopy (16). Proteins from these clusters are taken up in endosomal compartments in the upper epidermal layers. This endosomes remain perinuclear until removed with desquamation of keratinocytes. Due to depletion of desmosomes from DSG1 and absence of compensatory DSG3 in the subcorneal layer desmosomes will weaken and finally “melt” away here what leads to blistering. Future research has to address how previous observations on effects of PF serum on signal transduction and the activation of signal transduction molecules are connected to the ultrastructural findings. What does emerge from this and previous studies is that many factors have to be taken into consideration, which complicates such research. For instance why acantholysis only occurs when DSG3 is absent, meaning that whatever is the primary cause of acantholysis, steric hindrance, signal transduction or DSG1 depletion, neither one of them is able to induce acantholysis when DSG3 is still present. It is possible that clustering is a main cause of DSG1 depletion of desmosomes, but PF blisters can also be induced in organ culture in the absence of clustering (8, 10). Then for endocytosis of DSG1 it is unclear why it is absent in the basal layer, although endosomes are present here. Clustering starts in the basal layer as this is the first layer that encounters the pathogenic PF IgG, so something must be missing to start endocytosis.

## Data Availability Statement

All datasets generated for this study are included in the article/Supplementary Material.

## Author Contributions

DO, HP, and MJ contributed to the design of the study. DO, DK, and ES performed the experiments. DO and ES wrote the manuscript. HP and MJ revised the manuscript.

### Conflict of Interest

The authors declare that the research was conducted in the absence of any commercial or financial relationships that could be construed as a potential conflict of interest.
