# Supplementary material for: Endocytosis of IgG, Desmoglein 1, and Plakoglobin in Pemphigus Foliaceus Patient Skin
Source: Front Immunol. 2019 Nov 12;10:2635. doi: 10.3389/fimmu.2019.02635 (PMC6861377; doi:10.3389/fimmu.2019.02635)
Supplement: Supplementary file 1 [file Table_1.DOCX]

Supplementary Table 1: Suppliers of employed antibodies

| **Clone** | **Specificity** | **Supplier** |
| --- | --- | --- |
| DSG1-P23 | DSG1 ectodomain | PROGEN Biotechnik , Heidelberg, Germany |
| DSG1-P124 | DSG1 ectodomain | PROGEN Biotechnik , Heidelberg, Germany |
| 27B2 | DSG1 endodomain | Santa Cruz Biotechnology, Dallas, Texas, USA |
| 18D4 | DSG1 endodomain | Santa Cruz Biotechnology, Dallas, Texas, USA |
| DG3.10 | DSG1+2 endodomain | Acris Antibodies, Herford, Germany |
| B-11 | DSG1 endodomain | Santa Cruz Biotechnology, Dallas, Texas, USA |
| DSG-G194 | DSG3 endodomain | PROGEN Biotechnik , Heidelberg, Germany |
| U100 | DSC1 | PROGEN Biotechnik , Heidelberg, Germany |
| U114 | DSC3 | PROGEN Biotechnik , Heidelberg, Germany |
| 15F11 | PG | Sigma Aldrich, Saint Louis, Missouri, USA |
| DP2.15 | DP1+2 | PROGEN Biotechnik, Heidelberg, Germany |
| PKP3-270.6.2 | PKP3 | PROGEN Biotechnik , Heidelberg, Germany |
| 14/EEA1 | EEA1 | BD Transduction Laboratories, Franklin Lakes, New Jersey, USA |
| CTD-19 | CTS D | Santa Cruz Biotechnology, Dallas, Texas, USA |
| H4A3 | LAMP-1 | BioLegend, San Diego, California, USA |
| 4E6.2 | CNX43 | Abcam, Cambridge, UK |
